# Aggregation-Induced Emission Luminogen-Encapsulated Fluorescent Hydrogels Enable Rapid and Sensitive Quantitative Detection of Mercury Ions

**DOI:** 10.3390/bios13040421

**Published:** 2023-03-25

**Authors:** Wenchao Zhan, Yu Su, Xirui Chen, Hanpeng Xiong, Xiaxia Wei, Xiaolin Huang, Yonghua Xiong

**Affiliations:** 1 State Key Laboratory of Food Science and Technology, Nanchang University, Nanchang 330047, China; 2 School of Food Science and Technology, Nanchang University, Nanchang 330047, China; 3 Jiangxi-OAI Joint Research Institute, Nanchang University, Nanchang 330047, China

**Keywords:** functional hydrogels, AIEgens, Hg^2+^, chemosensor

## Abstract

Hg^2+^ contamination in sewage can accumulate in the human body through the food chains and cause health problems. Herein, a novel aggregation-induced emission luminogen (AIEgen)-encapsulated hydrogel probe for ultrasensitive detection of Hg^2+^ was developed by integrating hydrophobic AIEgens into hydrophilic hydrogels. The working mechanism of the multi-fluorophore AIEgens (TPE-RB) is based on the dark through-bond energy transfer strategy, by which the energy of the dark tetraphenylethene (TPE) derivative is completely transferred to the rhodamine-B derivative (RB), thus resulting in intense photoluminescent intensity. The spatial networks of the supporting hydrogels further provide fixing sites for the hydrophobic AIEgens to enlarge accessible reaction surface for hydrosoluble Hg^2+^, as well create a confined reaction space to facilitate the interaction between the AIEgens and the Hg^2+^. In addition, the abundant hydrogen bonds of hydrogels further promote the Hg^2+^ adsorption, which significantly improves the sensitivity. The integrated TPE-RB-encapsulated hydrogels (TR hydrogels) present excellent specificity, accuracy and precision in Hg^2+^ detection in real-world water samples, with a 4-fold higher sensitivity compared to that of pure AIEgen probes. The as-developed TR hydrogel-based chemosensor holds promising potential as a robust, fast and effective bifunctional platform for the sensitive detection of Hg^2+^.

## 1. Introduction

Heavy metals are one of the most toxic pollutants in the environment [1]. Among them, mercury and its compounds are reported to have extremely high toxicity. Moreover, mercury exists in many forms in nature, such as elemental mercury (Hg^0^), inorganic ions (Hg^2+^, Hg^+^) and organic compounds (CH_3_Hg^+^, C_2_H_5_Hg^+^) [2,3]. Among these forms, Hg^2+^ is considered to be a serious pollutant due to its stable existence in nature, widely existing in cosmetics, disinfectants, pharmaceuticals, and other industrial products [4,5,6]. Because of its strong affinity to sulfur-containing ligands, Hg^2+^ is easily coupled with biomacromolecules, such as enzymes and proteins [7,8,9]. Chronical exposure of organisms to mercury contamination may cause damage to the structure and function of internal organs, including dysfunction effects on the urinary [10,11,12], nervous [13,14], immune and reproductive systems [15,16,17]. To date, various instrumental analytical methods have been used to detect Hg^2+^ in aqueous solutions, such as atomic fluorescence spectrometry [18], inductively coupled plasma mass spectrometry [19], atomic absorption spectroscopy [20] and high-performance liquid chromatography [21]. Although these methods are sensitive and reliable, they depend on tedious sample preprocessing, expensive instruments, and skilled technicians, which is not suitable for rapid screening in the field.

Recently, fluorescent chemosensors with high sensitivity and fast response were proposed for Hg^2+^ recognition using fluorescent molecules as signal reporters, such as fluorescein isothiocyanate isomer (FITC) [22], rhodamine [7] and fluorescein [23]. However, these traditional luminescent materials with large conjugated coplanar molecular structures are prone to aggregation-caused quenching (ACQ) effect because of the strong π–π stacking interactions in concentrated solution [24,25]. Oppositely, AIEgens proposed by Tang’s group [26] in 2001 exhibit significantly enhanced fluorescence under aggregation state, which fundamentally overcomes the deficiency of the ACQ effect [27,28]. Since then, AIEgens have been widely used in fields of biochemical sensors [29,30,31], bioimaging [32,33,34,35] and nanomedicine [36,37]. However, in AIE-based chemosensors, the majority of hydrophobic AIEgens disperse in an aggregation state in aqueous solution, which largely restricts the reaction efficiency of AIEgens with hydrosoluble targets, such as ions [38]. Functional hydrogels are unique materials support with high water absorptivity and excellent film/fiber-forming capability [39,40,41]. The hydrogel fiber could serve as a rigid AIE-supporting network, as well create a hydrophilic space, allowing for efficient diffusion of water-soluble pollutants into the hydrogel matrix [42,43]. Moreover, the high loading efficiency and low optical background make hydrogels promising for ion adsorption and optical sensing [44,45].

Herein, we developed an AIEgen-encapsulated hydrogel probe for an ultrasensitive detection of Hg^2+^, by incorporating multi-fluorophore AIEgens into the agarose hydrogel matrix. Taking advantages of the dark through-bond energy transfer (DTBET) by connecting a Hg^2+^-sensitive rhodamine moiety with tetraphenylethene (TPE) derivative, the energy of the dark TPE derivative is completely transferred to the rhodamine-B derivative (RB) before non-radiative decay, which largely enhances the photoluminescence (PL) intensity of the AIEgens (TPE-RB). The hydrophobic AIEgens are fixed on the fibers of three-dimensional (3D) hydrogel networks, resulting in a strong fluorescence emission. Moreover, the abundant hydrogen bonds and the cross-linked 3D fiber network of hydrogel significantly promote the exchange between the hydrogel and the surrounding environment, which largely enriches the Hg^2+^ and provides a confined reaction space to facilitate the interactions between the TPE-RB and the Hg^2+^. The integrated TPE-RB-encapsulated hydrogels (TR hydrogels) presents significant superiority in minimizing manpower input by directly mixing with the pollutant samples and recording fluorescence signals through a high-throughput microplate reader. The results demonstrate that the TR hydrogel-based chemosensor shows excellent specificity for Hg^2+^, and the sensitivity is 4-fold higher that of pure AIEgen probes. In addition, the chemosensor exhibits high accuracy and precision in the spiked recovery experiments, verifying the high reliability of our proposed method. Taken together, the as-developed AIEgen-encapsulated hydrogels hold great potential as a simple, fast and effective bifunctional platform for the sensitive detection of Hg^2+^.

## 2. Materials and Methods

### 2.1. Materials and Reagents

Agarose and dimethyl sulfoxide (DMSO) were purchased from Sigma-Aldrich (St. Louis, MI, USA). TPE-RB was obtained based on a previous work [46]. Gel patterns were composed of porous micro-plates produced by 3D printing, and each channel was 6 mm in diameter and 10 mm in height. Concentrated nitric acid was purchased from Aladdin Reagent Co., Ltd. (Shanghai, China). Mercury dichloride (HgCl_2_), sodium chloride (NaCl), potassium chloride (KCl), zinc acetate ((CH_3_COO)_2_Zn), cupric sulfate (CuSO_4_), silver nitrate (AgNO_3_), nickel chloride hexahydrate puratrem (NiCl_2_·6H_2_O), cobalt chloride hexahydrate (CoCl_2_·6H_2_O), ferrous chloride (FeCl_2_), lead chloride (PbCl_2_) and iron chloride hexahydrate (FeCl_3_·6H_2_O) were purchased from Macklin Biochemical Co., Ltd. (Shanghai, China). All chemicals were analytical grade and used without further purification.

### 2.2. Instruments

Ultrapure water was obtained from a Milli-QA apparatus (Molsheim, France). The microwave oven was obtained from Midea Group Co., Ltd. (Hefei, China). The 96-well microplates were obtained from Corning Inc. (New York, NY, USA). Ultraviolet visible absorption spectra were measured on a Thermo Scientific NanoDrop 2000/2000C spectrophotometer (San Jose, CA, USA). Inductively coupled plasma atomic emission spectrometer optima 8000 (ICP-AES) was provided by Perkin Elmer Instrument Co., Ltd. (Norwalk, CT, USA). The Varioskan LUX multimode reader was purchased from Thermo Fisher Scientific (Waltham, MA, USA).

### 2.3. Synthesis of TR Hydrogel Probes

The TR hydrogel probes with a typical three-dimensional structure were synthesized according to a previously reported method [47]. Briefly, 15 mg agarose was dissolved in 4 mL of ultrapure water and oscillated evenly, and then the mixture was heated in the microwave medium for 2 min. After the agarose was completely dissolved, 1 μL of TPE-RB (5 mM, dissolved in DMSO) was added and homogenized. Subsequently, the mixture solution (120 μL) was added into the gel mold for solidification at 4 ℃ for 10 min, to prepare the TR hydrogel probes. As a control, a pure agarose hydrogel without TPE-RB molecules was also synthesized. To obtain the best detection results, several synthesizing parameters of the functional hydrogel, including the concentrations of agarose and TPE-RB, and the hydrogel volume were investigated and optimized.

### 2.4. Detection of Hg^2+^

For the detection of Hg^2+^, one piece of TR hydrogel was placed into 1.5 mL centrifuge tube containing 1 mL of standard solutions spiked with different Hg^2+^ concentrations, from 0 to 100 μM. After reacting for 40 min, the PL intensity of the reacted TR hydrogels was recorded at 595 nm using the Thermo Scientific Varioskan LUX multimode reader, under an excitation wavelength of 355 nm.

### 2.5. Real Sample Detection

The sewage samples were taken from a river near metalworking plants in Nanchang (Jiangxi, China) with simple pretreatment. In brief, 100 mL of sewage was taken, and then the insoluble substances were removed by centrifugation at 15,000× *g* rpm for 10 min. Subsequently, some nanoscale impurities were filtered by a filter membrane with a pore size of 220 nm. The concentration of Hg^2+^ was confirmed to be lower than 19.5 nM by ICP-AES. Then, 20 mL of the sewage supernatants were spiked by adding Hg^2+^ stock solution (5 mM) to produce a series of standard sample solutions with different Hg^2+^ concentrations. The obtained spiked sample solutions were finally measured by the proposed TR hydrogel-based chemosensor and ICP-AES.

The reliability and practicability of the developed TR hydrogel method was further evaluated by directly analyzing lake water samples from seven inland lakes in Nanchang City, including Qingshanhu Lake, Aixihu Lake, Yaohu Lake, Donghu Lake, Nanhu Lake, Meihu Lake and Xianghu Lake. The procedure of sample pretreatment and analysis of Hg^2+^ in lake water was the same as that of sewage samples. On this basis, these lake water samples were further spiked with the Hg^2+^ standard stock solution (5 mM) to obtain three different Hg^2+^-containing sample solutions with the Hg^2+^ concentration at 4 μM (Aixihu Lake and Donghu Lake), 6 μM (Meihu Lake and Yaohu Lake) and 7 μM (Nanhu Lake, Xianghu Lake and Qingshanhu Lake). The Hg^2+^ concentrations in these prepared spiked samples were then simultaneously determined by the designed TR hydrogels and the standard ICP-AES method.

## 3. Results and Discussion

### 3.1. Characterizations and Fluorescence Response of TR Hydrogels

**Scheme 1:**
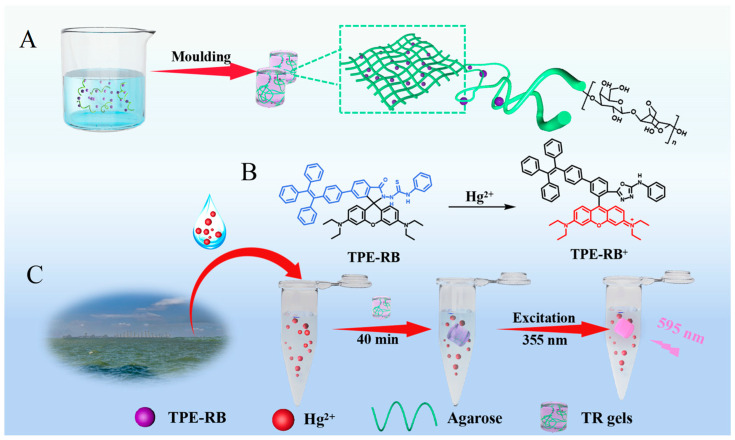
(**A**) Schematic illustration of the synthesis of TPE-RB-encapsulated agarose hydrogels. (**B**) Molecular structure change of TPE-RB probe after reacting with Hg^2+^. (**C**) Construction of the TR hydrogel chemosenor for Hg^2+^ contamination detection in actual samples.

**Figure 1:**
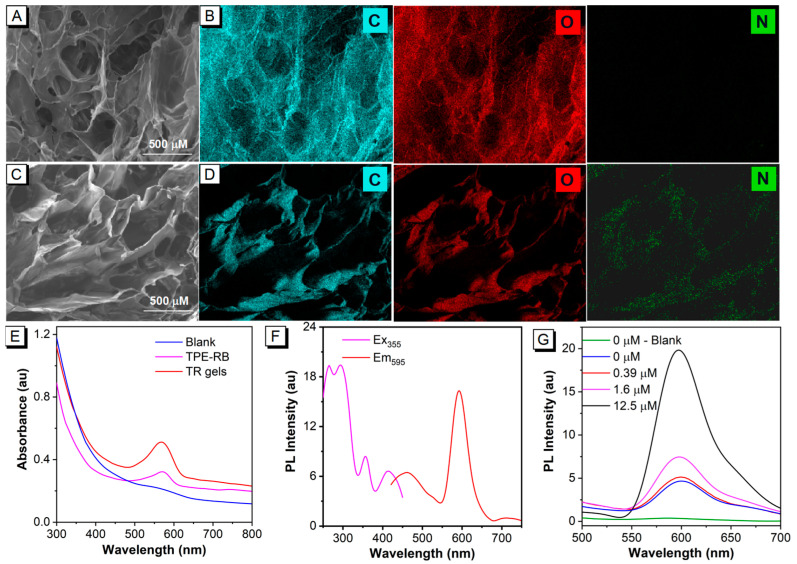
Characterizations of TR hydrogels. (**A**,**C**) SEM images and (**B**,**D**) EDX elemental-mapping images of agarose hydrogels and TR hydrogels. (**E**) UV-vis absorption spectra of agarose hydrogels, TPE-RB and TR hydrogels. (**F**) Excitation (Ex) and emission (Em) spectra of TR-FC hydrogel. (**G**) Fluorescent response of TR hydrogels in the presence of different concentrations of Hg^2+^. The blank represents pure-agarose hydrogels and the concentration of TPE-RB in TR hydrogels is 15 μM.

The TR hydrogels were prepared via one-pot integration of the agarose hydrosol and the TPE-RB dyes (Figure S1) in a 3D-printed mold (Scheme 1A, Figure S2). The TPE-RB is embedded in the fibrous network structure of the hydrogels formed from linear polysaccharide agarose. The d-galactose and 3,6-anhydro-L-galactose repeating units of agarose tend to assemble into random short-chain coils, by which the agarose solution is transformed to a hydrosol state at high temperature. Further, single and double helices are generated upon cooling, and then bundle and assemble into a fibrous network. The abundant hydroxyl groups in the agarose endow the hydrogel with excellent hydrophilicity to provide strong intramolecular and intermolecular hydrogen bond networks. Meanwhile, the AIEgens can be well wrapped in the crosslinking network. As shown in Figure 1A, scanning electron microscopy (SEM) image of lyophilized agarose hydrogels displays a macroporous and fluffy microstructure, indicating the abundant network of the hydrogels. The energy-dispersive X-ray (EDX) elemental-mapping images in Figure 1B show that C and O are present in the hydrogels. After AIEgen encapsulation, the TR hydrogels maintain the macroporous structure with a more compact fiber distribution (Figure 1C), and the EDX elemental-mapping images in Figure 1D demonstrate that the N element of the AIEgens is homogeneously distributed on the hydrogels, which verified that the hydrophobic TPE-RB AIEgens are well fixed on the agarose fiber networks and the successful fabrication of the TR hydrogels. Then, the optical properties of the as-prepared TR hydrogels were first investigated. Figure 1E shows that the prepared TR hydrogels preserve the characteristic absorption peak of TPE-RB at 570 nm. The multi-fluorophore TPE-RB presents an excitation and a maximum emission peak at 355 nm and 595 nm (Figure S3), respectively, displaying a large Stokes shift of about 240 nm. Such a large Stokes shift is highly conductive to avoid inner filter effect and interference between excitation and emission lights compared to the single RB molecule, which is usually excited under ~550 nm, with an extremely small Stokes shift. In addition, the fluorescence properties of TR hydrogels are well matched with the pure TPE-RB (Figure 1F). These results suggest that the attachment of AIEgens to 3D hydrogel has little effect on its photophysical properties.

Thereafter, the Hg^2+^-specific fluorescence response to TR hydrogels was studied. The detection principle of the TR hydrogels-based chemosensor depends on the specific recognition of TPE-RB to Hg^2+^ by the thiosemicarbazide group to form 1,3,4-oxadiazole. In the absence of Hg^2+^, the molecule presents a hydrophobic state, in which the RB derivative exhibits a non-emissive spirolactam form, and the TPE moiety emits blue fluorescence at 485 nm under 355 nm excitation. On the contrary, in the presence of Hg^2+^, the RB would undergo oxadiazole formation when the thiossemicarbazide moiety is liberated by Hg^2+^-facilitated ring opening of the spirocycle grouping. Meanwhile, the generated positively charged RB largely increases the solubility of the TPE-RB molecule in hydrogel. As a result, due to the energy transfer from TPE to rhodamine through DTBET, the TPE emission is weakened, and the fluorescence of rhodamine moiety will be intensified (Scheme 1B,C). The fluorescence signal changes of TR hydrogels after introduction of Hg^2+^ were monitored with the Hg^2+^ concentrations ranging from 0 μM to 12.5 μM. As shown in Figure 1G, increasing emission intensities of TR hydrogels were observed with the increase in Hg^2+^ concentration. In addition, the pure agarose hydrogels show negligible emission at 595 nm, indicating a low fluorescence background interference of the hydrogels.

### 3.2. Optimizations of the TR Hydrogel Formulation and Detection Conditions

**Figure 2:**
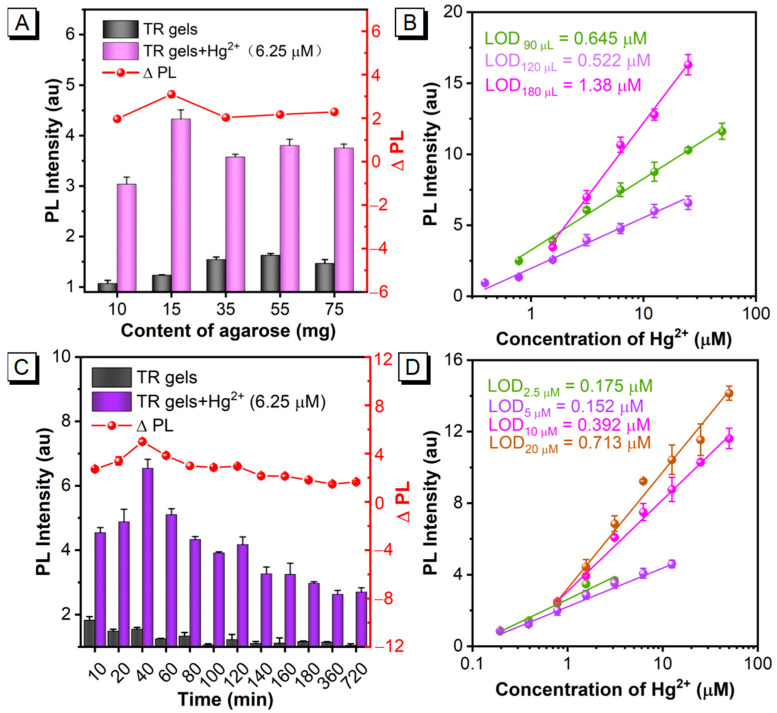
Optimizations of the TR hydrogels formulation and detection conditions. (**A**) The agarose feeding content, (**B**) volume, (**C**) reaction time and (**D**) AIEgen doping concentration of TR hydrogels chemosensor. The data are derived from the mean ± SD of three separate experiments.

To obtain the best detection performance of AIEgen-encapsulated hydrogel probes for Hg^2+^, several key parameters that could affect the sensing sensitivity were systematically explored and optimized, including the agarose content, hydrogel volume, reaction time and TPE-RB concentration. The agarose content in the reaction system was first optimized, since the aperture of agarose gel depends on the agarose concentration. As shown in Figure 2A, with the amount of agarose increasing from 10 mg to 75 mg, the background emission of the encapsulated hydrogels gradually increased, which may be due to the denser cross-linking network and smaller pores of the prepared hydrogels at the high agarose content, thus restricting the intramolecular motion of the TPE unit for high DTBET efficiency of the rhodamine moiety. Notably, after reacting with Hg^2+^ (6.25 μM), the high PL intensity with the maximum PL intensity difference (ΔPL) of the TR hydrogels was obtained when the agarose content was 15 mg. The possible reason is that the hydrogel probes with 10 mg of agarose present a limited confinement effect due to the relatively sparse network, while the TR hydrogels with the agarose feeding amount higher than 35 mg possess dense cross-linking networks and a lower water content, which may hinder the enrichment and dispersion of Hg^2+^ in the gel, resulting in lower ΔPL. Alongside that, we also found that the agarose solution could not be solidified into a complete hydrogel when the agarose content was less than 10 mg in the system, and the hydrogel formation speed was too rapid to blend well with the TPE-RB dyes to form the TR hydrogels when the agarose content was higher than 80 mg.

Subsequently, the volume of hydrogels was considered as another important factor that influences the sensitivity of the TR hydrogel-based chemosensor. Figure 2B and Figure S4 show that as the volume of the TR hydrogels increases from 90 μL to 120 μL, the limit of detection (LOD) decreases from 0.645 μM to 0.522 μM. However, further increasing the volume to 180 μL results in an increased LOD of 1.382 μM. Therefore, a gel volume of 120 μL was selected as the optimal fabrication condition for the TR hydrogel-based chemosensor, because of the moderate and available space for Hg^2+^ enrichment and reaction. In order to achieve a complete reaction of TR hydrogels with Hg^2+^, the reaction time was optimized. The results in Figure 2C indicate that the PL intensity of the TR hydrogels reached the maximum at 40 min and decreased gradually with further extension of reaction time. These results indicate that Hg^2+^ can fully diffuse into the TR hydrogels after 40 min to react with the TPE-RB molecules. However, excessive reaction time (>40 min) may cause the leakage of TPE-RB molecules into the solution, resulting in the reduction of the PL intensity of TR hydrogels. In order to investigate the stability of the prepared TR hydrogels in water, we monitored the changes in PL intensity of the supernatant and TR hydrogels within 40 min. As shown in Figure S5, the PL intensity of TR hydrogels and supernatant changed weakly, with an average of 1.75 and 0.08, respectively. The results indicated that the TR hydrogel probe had good stability in water, and the effect of AIEgen leakage on the detection performance of the hydrogel chemosensor was negligible. Therefore, 40 min was chosen as the optimal reaction time for the chemosensor. The concentration of TPE-RB in the TR hydrogels also influences the sensitivity of the developed chemosensor, because it is not only the fluorescence signal output unit, but also the response unit of Hg^2+^. High concentrations of TPE-RB dyes will cause increased background signal for a decreased signal-to-noise ratio and sensitivity, while low concentrations tend to reach reaction saturation and result in limited fluorescence response for narrow dynamic detection range. The results in Figure 2D and Figure S6 indicate that the lowest LOD value of 0.152 μM is achieved when the TPE-RB feeding amount is 5 μM. The LOD values of three other TR hydrogels with a TPE-RB feeding amount of 2.5, 10 and 20 μM, are 0.175 μM, 0.392 μM and 0.713 μM, respectively. Although the TR hydrogels with 2.5 μM and 5 μM TPE-RB show similar sensitivity, the hydrogels with the TPE-RB concentration of 5 μM present wider dynamic detection ranges, from 0.195 to 12.5 μM, compared with that of 2.5 μM (0.195–3.125 μM). These findings indicate that 5 μM TPE-RB was the optimal AIEgen concentration for the construction of the TR hydrogels. At the same time, the influence of solution pH on the chemosensor was investigated. The fluorescence response of TR hydrogels was monitored after reacting for 40 min by adding standard Hg^2+^ in aqueous solution with different pH values. As shown in Figure S7, the PL intensity of TR hydrogels decreased gradually with the increase of pH value in the solution, and the fluorescence dropped sharply after the pH value exceeded 8. Moreover, the pure-agarose hydrogels were used to verify that the hydrogel fiber network had a good adsorption effect on Hg^2+^. The results in Figure S8 show that the PL intensity of the solution decreased gradually with the increase in the hydrogel amounts in Hg^2+^ samples, indicating that TR hydrogels have advantages as bifunctional chemosensors for Hg^2+^ detection and adsorption in water.

### 3.3. Analytical Performance of the TR Hydrogel Chemosensor

**Figure 3:**
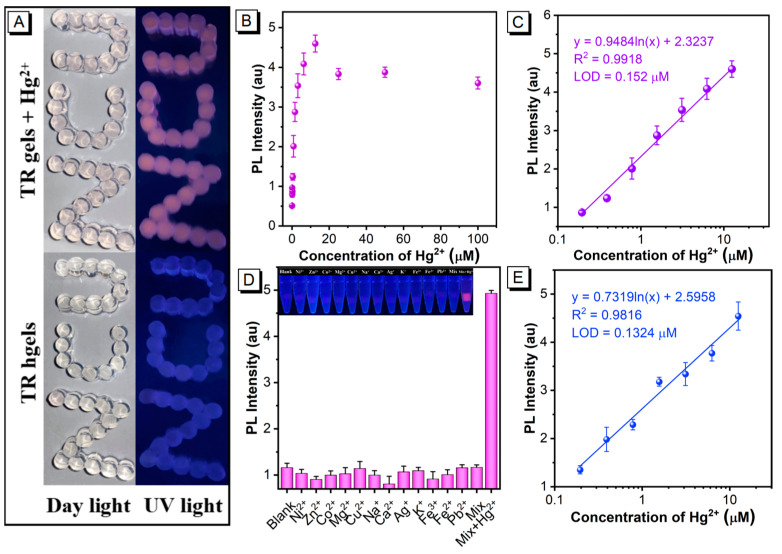
(**A**) Photos of TR hydrogels before and after reacted with Hg^2+^ under daylight and ultraviolet light. (**B**) PL intensity of TR hydrogel chemosensor after reacted with Hg^2+^, with the concentrations ranging from 0 μM (as a negative control) to 100 μM. (**C**) Linear plot of PL intensity of TR hydrogels against the logarithm of Hg^2+^ concentration. (**D**) Cross-reactivity of TR hydrogels to interferential metal ions. The concentrations of Hg^2+^ and other metal ions are 6.25 μM and 25 μM, respectively. Inset is the photo of TR hydrogels under ultraviolet light after reacting with different metal ions. (**E**) Quantitative calibration curve of Hg^2+^ in real-world water, using the TR hydrogel chemosensor. The Hg^2+^-spiked standard solution is real-world water with Hg^2+^ concentrations ranging from 0 μM to 100 μM. The data are derived from the mean ± SD of three separate experiments.

Based on the above investigations, the optimal TR hydrogels were manufactured for Hg^2+^ detection. Figure 3A shows that the TR hydrogels were lined up as “NCU” (abbreviation of Nanchang University) and the “NCU” is transparent under daylight and emits blue fluorescence under ultraviolet light. After the TR hydrogels reacted with the Hg^2+^ solution (6.25 μM) for 40 min, the “NCU” appears reddish under daylight and exhibits red emission under ultraviolet light. This is because the TPE-RB shows blue fluorescence (~485 nm) of aggregated TPE due to its strong hydrophobicity. When the TPE-RB reacted specifically with Hg^2+^ in the 3D hydrogel network, the solubility of rhodamine groups in water will be improved, and the energy of TPE moiety will be transferred to rhodamine through the DTBET process under ultraviolet light, resulting in intense red emission. These results demonstrate that the TR hydrogels can provide an intuitive visual result, indicating the presence of Hg^2+^. Encouraged by this, the fluorescence response of the proposed TR hydrogels to different concentrations of Hg^2+^ (0–100 μM) was further investigated. The result in Figure 3B shows that the PL intensity gradually increases with increasing Hg^2+^ concentration, from 0 μM to 100 μM. By plotting the standard curve between the PL intensity and the logarithm of Hg^2+^ concentrations, the developed TR hydrogel sensor exhibits an excellent linear detection range, from 0.195 μM to 12.5 μM, with a correlation coefficient (R^2^) of 0.9918 (Figure 3C). The regression equation is represented by *y* = 0.9484 ln(*x*) + 2.3237, where *y* is the PL intensity and *x* is the Hg^2+^ concentration. The LOD, which is defined as the Hg^2+^ concentration corresponding to the mean value of 20 negative samples plus three-fold standard deviation, was calculated to be 0.152 μM. At the same time, the detection performance of the TPE-RB probe was compared. Figure S9 displays a relatively narrow linear relationship between the PL intensity and the Hg^2+^ concentration, from 0.781 μM to 3.125 μM, with an R^2^ of 0.987 and an LOD of 0.623 μM, which was about 4-fold higher than that of the TR hydrogel chemosensor. These results reveal that the smart integration of AIEgens with hydrogels can effectively improve the fluorescent response sensitivity of the TPE-RB molecule against Hg^2+^ in aqueous solution. We speculate that the possible reason is that Hg^2+^ can diffuse and leach into the agarose fiber network due to the abundant hydrogen bonds in hydrogels, which could effectively improve the reaction efficiency with dyes by enriching metal ions in the confined space owing to the confinement effect of 3D hydrogel networks, thereby improving the analytical sensitivity. Compared with other Hg^2+^ fluorescence sensors (Table S1), our proposed hydrogel chemosensor shows good detection performance and can be used as a bifunctional platform for Hg^2+^ detection and removal. To further investigate the selectivity of the developed assay, the fluorescence response of the TR hydrogels to other common metal ions, including Ni^2+^, Zn^2+^, Co^2+^, Mg^2+^, Cu^2+^, Na^+^, Ca^2+^, Ag^+^, K^+^, Fe^3+^, Fe^2+^, Pb^2+^, and their mixtures with Hg^2+^, were determined. Figure 3D suggests that negligible changes in the PL intensity at 595 nm were observed for these interfering ions and their mixture, proving its good sensing selectivity toward Hg^2+^ and excellent anti-interference ability.

### 3.4. Quantitative Detection of Hg^2+^ in Real-World Water Samples

Encouraged by the satisfying Hg^2+^-sensing performances of the developed TR hydrogels chemosensor, we further evaluated its potential to determine Hg^2+^ in real-world sewage and lake water samples. Prior to the measurement, these two complex water samples were first filtered. Figure 3E shows the quantitative standard curve of the TR hydrogel chemosensor for Hg^2+^ in sewage, in which the PL intensity increases as the Hg^2+^ concentration increases, with an excellent linear relationship observed at the Hg^2+^ concentration of 0.195 μM to 12.5 μM, and an R^2^ of 0.982. The LOD was calculated to be 0.132 μM according to the above methods. Subsequently, the quantitative accuracy and precision of the TR hydrogels for Hg^2+^ in sewage were evaluated by analyzing five different Hg^2+^-spiked samples with the Hg^2+^ concentration at 0.5 μM, 1 μM, 4 μM, 6 μM and 8 μM. Table 1 shows that the average recoveries of Hg^2+^ in sewage range from 82.54% to 109.59%, with the coefficient of variation (CV) ranging from 2.51% to 12.17%, indicating an acceptable accuracy and precision for Hg^2+^ quantification via the TR hydrogel-based chemosensor. Furthermore, lake water samples were collected from seven freshwater lakes in Nanchang City, including Qingshanhu Lake, Aixihu Lake, Yaohu Lake, Donghu Lake, Nanhu Lake, Meihu Lake and Xianghu Lake (Figure S10). ICP-AES analysis showed that the Hg^2+^ concentrations were lower than 19.5 nM in all the seven freshwater lakes (Table S2), which is out of the quantitative range of our proposed TR hydrogels. Therefore, we chose them as real-world samples to evaluate the reliability of our method by spiking 4 μM, 6 μM and 7 μM Hg^2+^ into these lake water samples. These spiked lake water samples were then simultaneously measured by the ICP-AES method. The results in Table 2 indicate that the average recoveries of the instrumental method and our proposed method are 94.71–119.5% and 86.45–109.22%, respectively, implying that the two methods have good correspondence in the analysis of lake water samples. The above results demonstrated that the proposed TR hydrogel-based chemosensors can serve as ideal candidates for real-world Hg^2+^ quantification, with a negligible matrix effect.

## 4. Conclusions

In summary, we successfully developed a TR hydrogel probe by incorporating the multi-fluorophore TPE-RB AIEgens with agarose hydrogel for the sensitive detection of Hg^2+^ in water. The 3D fiber network structure of hydrogel can effectively fix the hydrophobic AIEgens and enlarge accessible reaction surface for hydrosoluble Hg^2+^. Moreover, the hydrogel presents superior capacity for adsorbing Hg^2+^ in water, and provides a confined 3D reaction space, which is helpful to improve the reaction efficiency between Hg^2+^ and TPE-RB, thereby enhancing the sensitivity. Compared with the TPE-RB probe, the proposed TR hydrogels exhibit higher sensitivity with a 4-fold lower LOD. The accuracy and reproducibility of the proposed method were also compared with those of the standard method. Collectively, the TR hydrogel probe has dual functional advantages of simultaneously detecting and absorbing heavy metals, and can be used as a promising chemosensing platform for rapid field screening of heavy metal ions in complex samples.

## Figures and Tables

**Table 1:** Precision analysis of the developed TR hydrogel chemosensor in Hg^2+^-spiked sewage water samples.

Spiking Levels (μM)	Mean (μM)	CV (%)	Recovery (%)
0.5	0.55±0.25	12.17	109.59
1	0.83±0.24	10.30	82.54
4	4.12±0.31	7.06	103.03
6	5.16±0.17	4.60	86.02
8	7.30±0.10	2.51	91.29

**Table 2:** Analysis of Hg^2+^ contamination in spiked lake samples by ICP-AES and TR-hydrogel methods.

Lake	Spiking Levels (μM)	ICP-AES		TR Hydrogel Chemosensor		
		Mean (μM)	Recovery (%)	Mean (μM)	Recovery (%)	CV (%)
Aixihu Lake	4	4.78	119.5	3.81	95.21	8.21
Donghu Lake	4	4.78	119.5	3.43	86.45	4.64
Meihu Lake	6	6.26	104.33	6.56	109.22	5.12
Yaohu Lake	6	6.26	104.33	5.61	94.63	7.99
Nanhu Lake	7	6.63	94.71	7.13	102.72	8.73
Xianghu Lake	7	6.63	94.71	6.69	99.85	2.37
Qingshanhu Lake	7	7.37	105.29	7.35	105.18	5.11

## Data Availability

The data that support the findings of this study are available from the corresponding author upon reasonable request.

## Supplementary Materials

The following supporting information can be downloaded at: https://www.mdpi.com/article/10.3390/bios13040421/s1, Figure S1: Molecular structure of TPE-RB; Figure S2: Schematic image of a 3D-printed mold for synthesizing TR hydrogels; Figure S3: Fluorescence excitation spectrum and emission spectrum of TPE-RB; Figure S4: Volume optimization of TR hydrogels; Figure S5: The PL intensity of the supernatant and TR hydrogels within 40 min; Figure S6: Quantitative assay for Hg^2+^ in ultrapure water, using the TR hydrogel chemosensor; Figure S7: Effect of pH on fluorescence response of the TR hydrogel chemosensor; Figure S8: PL intensity of TPE-RB in Hg^2+^-spiked water after Hg^2+^ was adsorbed by different numbers of agarose hydrogels; Figure S9: Quantitative calibration curve of Hg^2+^ using TPE-RB probes; Figure S10: Locations of the sampling sites of the freshwater lakes in Nanchang City, Jiangxi Province; Table S1: Comparison of the sensitivities and linear ranges of different fluorescent sensors for Hg^2+^ detection; Table S2: Analysis of Hg^2+^ contamination in real lake samples by ICP-AES and TR hydrogels methods [48,49,50,51,52,53,54,55,56].
