# Supplementary material for: Aggregation-Induced Emission Luminogen-Encapsulated Fluorescent Hydrogels Enable Rapid and Sensitive Quantitative Detection of Mercury Ions
Source: Biosensors (Basel). 2023 Mar 25;13(4):421. doi: 10.3390/bios13040421 (PMC10135736; doi:10.3390/bios13040421)
Supplement: Supplementary file 1 [file biosensors-13-00421-s001.zip › biosensors-2286421-supplementary.pdf]

Supporting Information for:

Aggregation-induced emission luminogens-encapsulated fluorescent hydrogels enable rapid and sensitive quantitative detection of mercury ions

Wenchao Zhan[1,2#], Yu Su[1,2#], Xirui Chen[1,2], Hanpeng Xiong[1,2], Xiaxia Wei[1,2], Xiaolin Huang[1,2,3*], and Yonghua Xiong[1,2,3*]

[1] State Key Laboratory of Food Science and Technology, Nanchang University, Nanchang 330047, P. R. China

[2] School of Food Science and Technology, Nanchang University, Nanchang 330047, P. R. China

[3] Jiangxi-OAI Joint Research Institute, Nanchang University, Nanchang 330047, P. R. China

[#]These authors contributed equally to this work.

*Correspondence to:

Dr. Xiaolin Huang and Dr. Yonghua Xiong

E-mail: hxl19880503@163.com (X. H.); yhxiongchen@163.com (Y. X.)

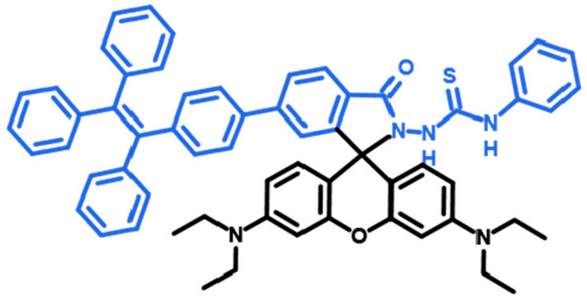

TPE-RB

Figure S1. Molecular structure of TPE-RB.

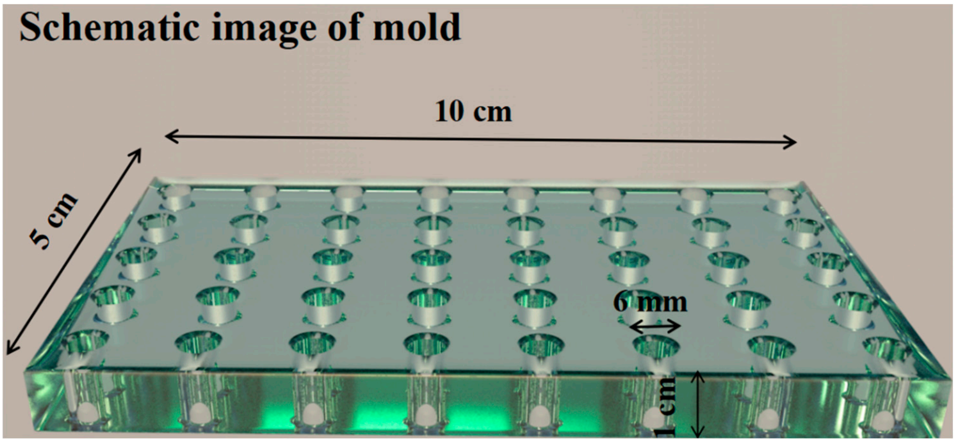

Figure S2. Schematic image of a 3D-printed mold for synthesizing TR hydrogels.

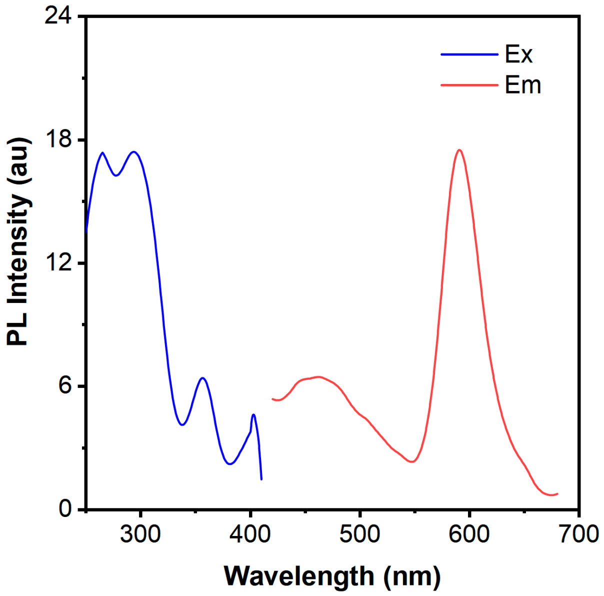

Figure S3. Fluorescence excitation spectrum and emission spectrum of TPE-RB.

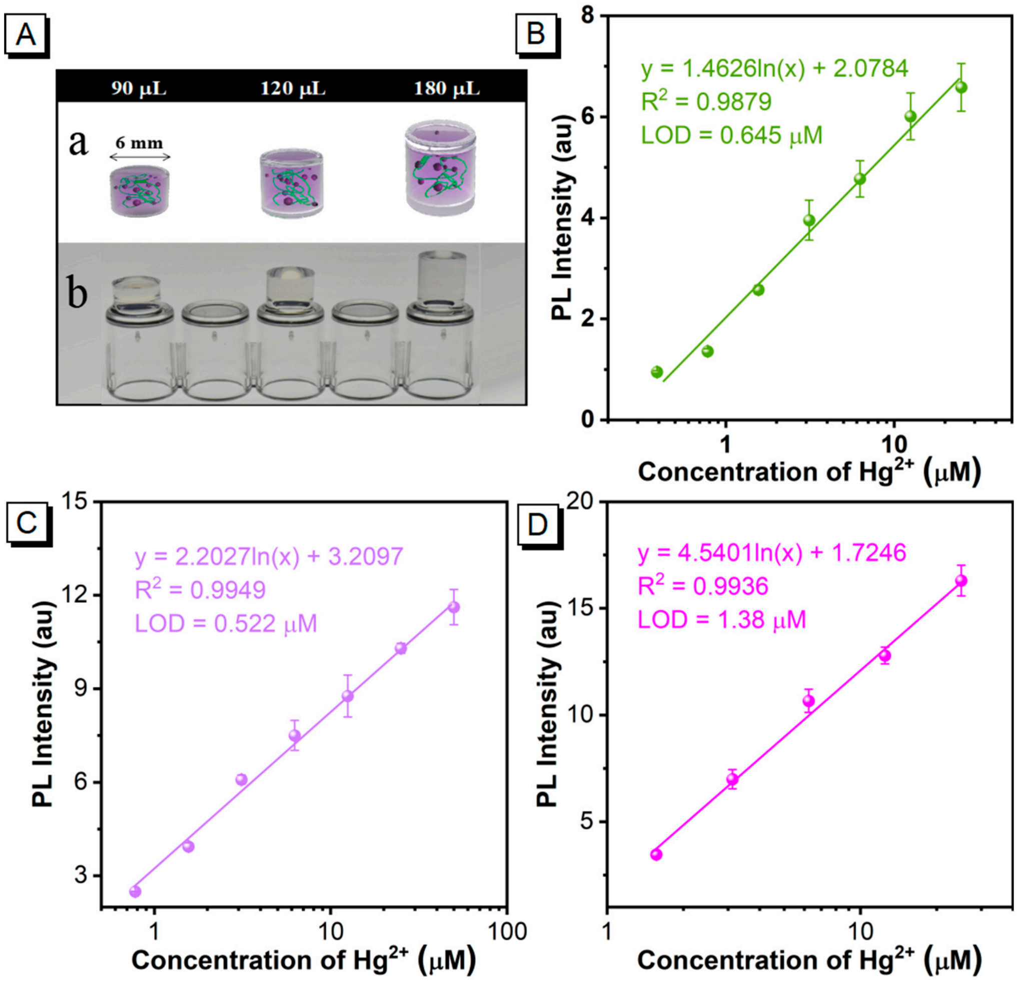

Figure S4. Volume optimization of TR hydrogels. (A) (a) Schematic and (b) actual images of TR hydrogels with different molding volumes of 90 μL, 120 μL and 180 μL. Quantitative assay for Hg^{2+} in ultrapure water using TR hydrogels with molding volumes of (B) 90 μL, (C) 120 μL and (D) 180 μL. The data are derived from the mean ± SD of three separate experiments.

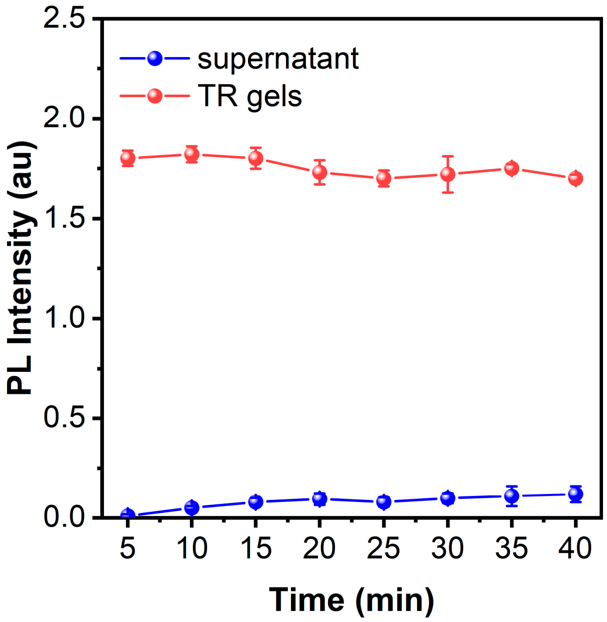

Figure S5. The PL intensity of the supernatant and TR hydrogels within 40 min.

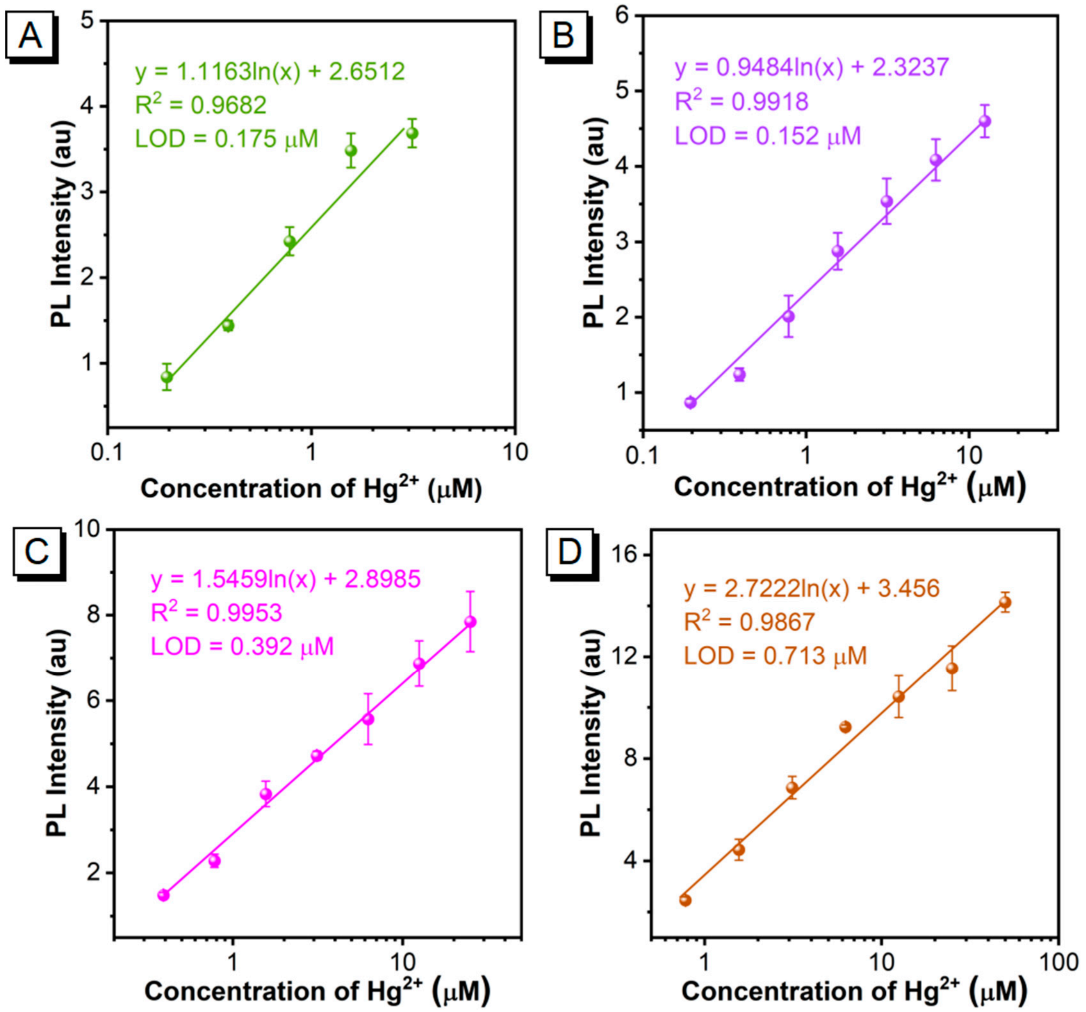

Figure S6. Quantitative assay for Hg^{2+} in ultrapure water using TR hydrogels chemosensor prepared by mixing (A) 2.5 μM, (B) 5 μM, (C)10 μM, (D) 20 μM of TPE-RNS in 120 μL of hydrogel. The data are derived from the mean ± SD of three separate experiments.

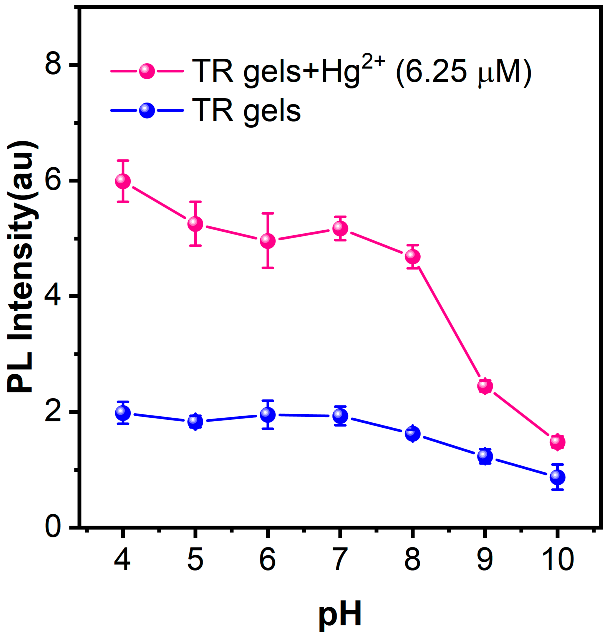

Figure S7. Effect of pH on fluorescence response of TR hydrogel chemosensor.

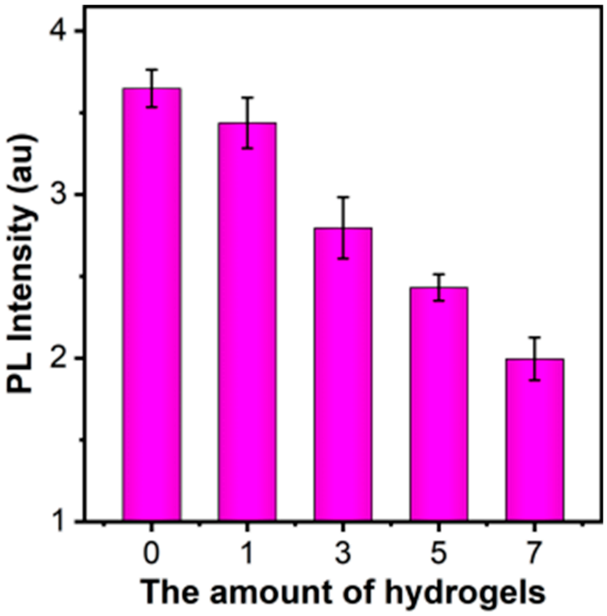

Figure S8. PL intensity of TPE-RB in Hg^{2+}-spiked water after Hg^{2+} was adsorbed by different amounts of agarose hydrogels.

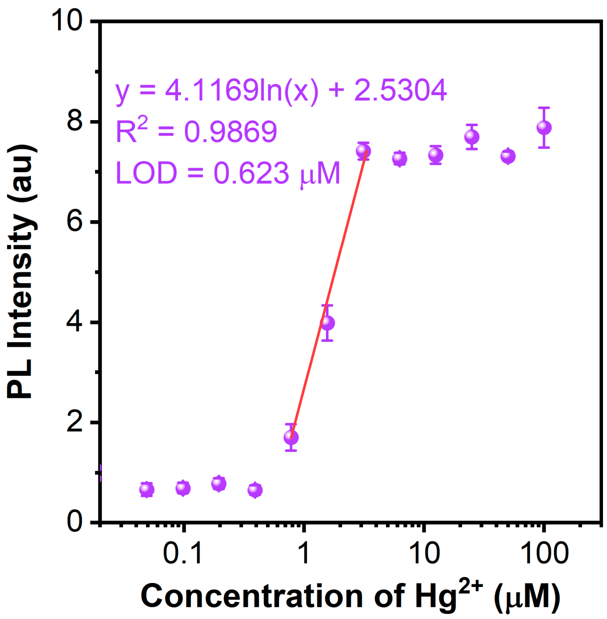

Figure S9. Quantitative calibration curve of Hg^{2+} using TPE-RB probes with spiked-Hg^{2+} concentrations ranging from 0 μM to 100 μM. The data are derived from the mean ± SD of three separate experiments.

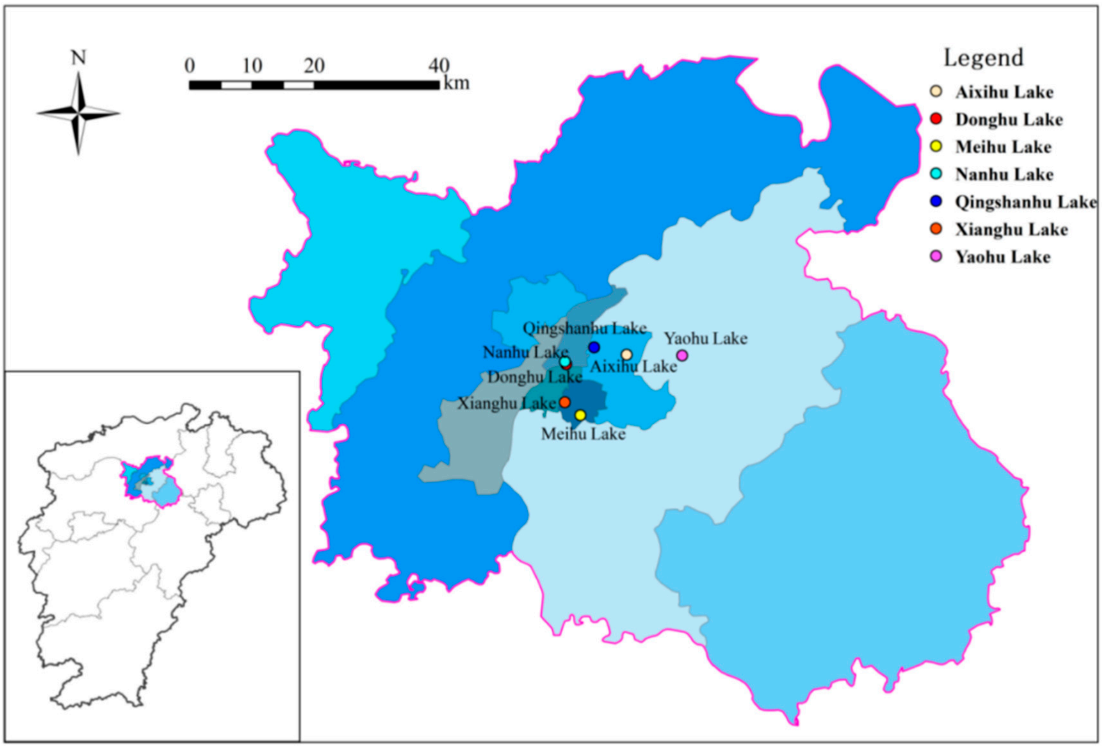

Figure S10. Locations of the sampling sites of the freshwater lakes in Nanchang City, Jiangxi Province.

Table S1. Comparison of the sensitivities and linear ranges of different fluorescent sensors for Hg^{2+} detection.

No.	System	Linear range (μM)	LOD (μM)	Reference
1	RTTU sensor	25-200	0.304	[1]
2	RBTP chemosensor	0-4	0.27	[2]
3	Cellulose-g-RD	-	50	[3]
4	PPT-membrane	-	1.6	[4]
5	Isatin-based chemosensor	-	2.5	[5]
6	HL fluorescent sensor	0.5-4	0.26	[6]
7	PVA-hydrogel films	0-1000 ppb	2 ppb	[7]
8	Zn-MOF	0-1200	0.32	[8]
9	Hydrogel sensors	-	0.01	[9]
10	TR hydrogels	0.195-12.5	0.152	This work

Table S2. Analysis of Hg^{2+} contamination in real lake samples by ICP-AES and TR hydrogel methods.

Lake	ICP-AES (nM)	TR hydrogels chemosensor (nM)
Aixihu Lake	2.03	-
Donghu Lake	< 0.737	-
Meihu Lake	< 0.737	-
Yaohu Lake	< 0.737	-
Nanhu Lake	< 0.737	-
Xianghu Lake	< 0.737	-
Qingshanhu Lake	19.5	-

"-" means not detected.

Reference

1. M. Hong, S. Lu, F. Lv, D. Xu, A novel facilely prepared rhodamine-based Hg^{2+} fluorescent probe with three thiourea receptors, *Dyes Pigm.* **2016**, *127*, 94-99. http//10.1016/j.dyepig.2015.12.023
2. S. Chen, W. Wang, M. Yan, Q. Tu, S.-W. Chen, T. Li, M.-S. Yuan, J. Wang, 2-Hydroxy benzothiazole modified rhodol: aggregation-induced emission and dual-channel fluorescence sensing of Hg^{2+} and Ag^{+} ions, *Sens. Actuators, B* **2018**, *255*, 2086-2094. http//10.1016/j.snb.2017.09.008
3. L.Q. Xu, K.-G. Neoh, E.-T. Kang, G.D. Fu, Rhodamine derivative-modified filter papers for colorimetric and fluorescent detection of Hg^{2+} in aqueous media, *J. Mater. Chem. A* **2013**, *1*. http//10.1039/c2ta01072k
4. S. Hussain, S. De, P.K. Iyer, Thiazole-containing conjugated polymer as a visual and fluorometric sensor for iodide and mercury, *ACS Appl. Mater. Interfaces* **2013**, *5*, 2234-40. http//10.1021/am400123j
5. G. Mohammadi Ziarani, S. Roshankar, F. Mohajer, A. Badiei, M. Sillanpää, The synthesis of SBA-Pr-N-Is-Bu-SO3H as a new Hg^{2+} fluorescent sensor, *Inorg. Chem. Commun.* **2022**, *146*. http//10.1016/j.inoche.2022.110100
6. H.-L. Wu, J.-P. Dong, F.-G. Sun, R. Li, Y.-X.J.J.o.A.S. Jiang, New Selective Fluorescent "Turn-On" Sensor for Detection of Hg^{2+} Based on a 1, 8-Naphthalimide Schiff Base Derivative, *J. Appl. Spectrosc.* **2022**, *89*, 487-494.
7. S. Wu, Y. Yang, Y. Cheng, S. Wang, Z. Zhou, P. Zhang, X. Zhu, B. Wang, H. Zhang, S. Xie, Z. Zeng, B.Z. Tang, Fluorogenic detection of mercury ion in aqueous environment using hydrogel-based AIE sensing films, *Aggregate* **2022**. http//10.1002/agt2.287
8. J. Xiao, J. Liu, X. Gao, G. Ji, D. Wang, Z. Liu, A multi-chemosensor based on Zn-MOF: Ratio-dependent color transition detection of Hg (II) and highly sensitive sensor of Cr (VI), *Sens. Actuators, B* **2018**, *269*, 164-172. http//10.1016/j.snb.2018.04.129
9. Z. Qu, C. Wang, H. Duan, L. Chi, Highly efficient and selective supramolecular hydrogel sensor based on rhodamine 6G derivatives, *RSC Adv.* **2021**, *11*, 22390-22397. http//10.1039/d0ra10890a
